# Dr. T. E. A.

**Published:** 1875-08

**Authors:** 


*Dr. T. E. A.*

If you are right you need have no fears. If you are wrong retrace your steps as soon as possible. All dishonorable means used to make wrong appear right, will only serve to steep you more indelably in infamy. If your friends are so constituted as not to be able to adhere to the truth pity them; they can do you no harm. Stand firm, and you will be an honor to yourself and to the God who made you, whilst those who are governing alone from policy will lose their self respect—the respect of all truly honorable men. P.

